# Navigating the Science System: Research Integrity and Academic Survival Strategies

**DOI:** 10.1007/s11948-024-00467-3

**Published:** 2024-04-03

**Authors:** Andrea Reyes Elizondo, Wolfgang Kaltenbrunner

**Affiliations:** Centre for Science and Technology Studies, Leiden University, Leiden, The Netherlands

**Keywords:** Research integrity, Questionable research practices, Epistemology, Labor, Research culture

## Abstract

Research Integrity (RI) is high on the agenda of both institutions and science policy. The European Union as well as national ministries of science have launched ambitious initiatives to combat misconduct and breaches of research integrity. Often, such initiatives entail attempts to regulate scientific behavior through guidelines that institutions and academic communities can use to more easily identify and deal with cases of misconduct. Rather than framing misconduct as a result of an information deficit, we instead conceptualize Questionable Research Practices (QRPs) as attempts by researchers to reconcile epistemic and social forms of uncertainty in knowledge production. Drawing on previous literature, we define epistemic uncertainty as the inherent intellectual unpredictability of scientific inquiry, while social uncertainty arises from the human-made conditions for scientific work. Our core argument—developed on the basis of 30 focus group interviews with researchers across different fields and European countries—is that breaches of research integrity can be understood as attempts to loosen overly tight coupling between the two forms of uncertainty. Our analytical approach is not meant to relativize or excuse misconduct, but rather to offer a more fine-grained perspective on what exactly it is that researchers want to accomplish by engaging in it. Based on the analysis, we conclude by proposing some concrete ways in which institutions and academic communities could try to reconcile epistemic and social uncertainties on a more collective level, thereby reducing incentives for researchers to engage in misconduct.

## Introduction

Research Integrity (RI) is high on the agenda of both institutions and science policy (ALLEA, 2017; ENRIO, 2019; Evroux, 2022; Hastings et al., 2022; WCRI statements, 2010, 2013). The European Union as well as national ministries of science have launched ambitious initiatives to combat research misconduct and breaches of RI. Often, such initiatives entail attempts to regulate scientific behavior through guidelines that institutions and academic communities can use to more easily identify and handle cases of misconduct. For example, the Horizon project PRINTEGER (Promoting Integrity as an Integral Dimension of Excellence in Research, 2015) resulted in a policy brief for science policy makers and research managers, a policy brief for scientific and scholarly publishers, and a web-based educational tool called Upright (Juurik et al., 2018; Leeuwen et al., 2018; Upright, 201^2018^8). Another EU project, SOPs4RI (Standard Operating Procedures for Research Integrity, 2018). Another EU project, SOPs4RI (Standard Operating Procedures for Research Integrity), created a collection of guidelines and standard operating procedures that universities can readily implement to promote research integrity (Toolbox for Research Integrity, 2022).

While we commend their solution-oriented approach, we argue in this paper that such ongoing initiatives can benefit from a more detailed conceptual analysis of the rationales that underpin questionable research practices (QRPs). One implication of the above approaches is in fact the assumption that outright misconduct and QRPs are analytically relatively uninteresting, in the sense that they simply constitute a case of individuals failing to live up to community standards of ethical behavior.

We instead propose to conceptualize QRPs as in some ways characteristic of and endemic to academic research practices embedded in a particular economic and social system. More specifically, we will argue that integrity issues can be usefully analyzed as a special strategy to reconcile the tensions between different forms of uncertainty in scientific work and academic employment. While this might at first sight be read as a form of relativizing or excuse of misconduct, we believe that such an analysis is crucial for understanding the concrete underlying rationales, thereby also providing better tools for institutions and academic communities to prevent QRPs.

Breaches of RI are empirically notoriously hard to study (Antonakaki, 2019; Levelt et al., 2012). They can usually not be observed as they unfold and tend to be shrouded in outrage and shame when uncovered. The analysis we present in this paper is based on empirical material from 30 focus group interviews carried out with researchers, funders, and other stakeholders from different fields and across a range of European countries (organized in the context of the above mentioned SOPs4RI project). The conversations inter alia created opportunities for researchers to openly discuss the rationales for misconduct and QRPs. Mirroring our analytical perspective, their accounts often described misconduct as part of a spectrum of more widely shared practices that span various degrees of acceptability within a community. As such, they also shed light on the underlying systemic problems in academic research that misconduct is often a (however reproachable and ultimately counterproductive) solution to.

The paper is organized as follows. In the next section, we lay out our conceptual framework and position it against some previous literature. We then discuss in more detail the specificities of our data collection and coding of the data. In the subsequent empirical section, we discuss various overarching “families” of QRPs. We argue that different types of misconduct can be grouped together because they share some principal underlying strategies for reconciling intersecting social and epistemic constraints on daily research practice. In conclusion, we discuss what our findings mean for institutional and policy attempts to fight research misconduct.

## Conceptual Framework

A significant part of previous research on scientific misconduct has been concerned with categorizing misconduct and with interrogating the incidence of different forms of misconduct (Bouter et al., 2016; Fanelli, 2009, 2011), as well as with the specificity of certain forms in particular fields (John et al., 2012; Ravn & Sørensen, 2021). Research has also focused on how to promote RI both by Research Performing Organizations (RPOs), Research Funding Organizations (RFOs), and scientific journals (Bouter, 2020; Labib et al., 2021; Mejlgaard et al., 2020; Moher et al., 2020; Sørensen et al., 2021; Steneck, 2006). Drawing on the insights resulting from these efforts, initiatives have been developed that highlight the importance of good practices as an integral part of a culture of integrity (Collaborative Working Group from the Conference Keeping the Pool Clean: Prevention and Management of Misconduct Related Retractions, 2018; CSE, 2021; Forsberg et al., 2018; Global Science Forum, 2007; Tri-Agency Framework on Responsible Conduct of Research, 2021).

Other research has focused on the role that motivations and systemic incentives play in the incidence of RI breaches (Bruton et al., 2020; Sacco et al., 2019). In our own approach to RI, we take this approach further by highlighting the relation between misconduct and various forms of uncertainty, which is in several ways a key characteristic of scientific practice. There is on one hand the inherent uncertainty of scientific inquiry. Science is the interrogation of the unknown, which constitutes a perpetual source of unpredictability. A typical manifestation is the risk of a “failed” experiment, in the sense that the experiment turns out not to be realizable due to practical or technical problems, or in the sense that it does not produce interesting results that go beyond what is already known. Following Sigl (2016), we propose to call this type of uncertainty epistemic uncertainty, since it directly relates to the inherent underdetermination of scientific and scholarly inquiry, be it in laboratory settings, observational research, or more interpretive scholarship.

In addition, following Sigl (2016) there is also social uncertainty, by which we mean unpredictability that arises from the human-made conditions for scientific work. Scholarly and scientific inquiry is embedded in concrete practical circumstances that heavily affect researchers’ ability to engage with research questions. This includes the relative ease with which researchers can access resources such as funding, labor, instruments and research infrastructure. Many observers agree that the social uncertainty of research thus conceptualized has increased in recent decades. In many countries and fields, the share of short term contracts of researchers has grown disproportionally, while the degree of competition for funding has increased (Fochler & Sigl, 2018; Whitley et al., 2010). In addition, many countries and institutions have adopted evaluative practices that reward individuals on the basis of their publication output and citations instead of focusing on the output and results of a research team (Müller, 2014).

A crucial question is how epistemic and social uncertainty relate to each other. Critical observers propose that a main problem in contemporary science systems is that they are too tightly coupled (Hackett, 1987; Laudel, 2006; Sigl, 2016). For example, an experiment that fails to deliver “exciting” data can become an existential problem for individual researchers, because it prevents them from publishing articles and thus from acquiring more funding (Matosin et al., 2014; Teixeira da Silva, 2015). In a system where epistemic and social uncertainty are tightly coupled, a failed experiment can thus mean the immediate end of a career. Many have argued that such tight coupling makes researchers risk averse and ultimately stifles scientific creativity (Zoller et al., 2014).

As a growing body of literature has documented, researchers have developed strategies to soften the tight coupling of epistemic and social uncertainty (Fochler & Sigl, 2018; Hackett, 1987; Kaltenbrunner, 2020; Sigl, 2016). The range of strategies described in this literature varies in terms of how individual/collective they are. For example, some strategies are built around a principle of creating resource buffers on the level of research groups and thus have a more collective outlook. Others are primarily meant to limit the engagement of researchers in activities that threaten their opportunities in academic career systems or promise only limited individual benefits.

In this paper, we are interested in the implications of such strategies for RI. We will illustrate types of behavior that contradict “good scientific practice”, not primarily with malign intent but because they allow for a loosening of the coupling of epistemic and social uncertainties (thereby reducing risks to careers and the continuation of research). The empirical material constitutes a spectrum of ways to achieve such loosening. It includes, on one hand, practices to pre-emptively decrease the epistemic uncertainty of research, for example by avoiding occasions where the robustness of research findings becomes visible and could be questioned. Other strategies mainly focus on reducing social uncertainty. We will for example discuss grant acquisition strategies geared to maximize the likelihood of success even at the risk of sacrificing what researchers otherwise consider good research practice. Yet another manifestation are practices of avoiding the head-on tackling of research integrity issues where it involves risks for career development. In all cases, then, researchers ultimately aim to reduce one of the two forms of uncertainty so as to create an overall more favorable balance between them. As will become clear from the analysis, some of the behaviors studied are universally considered outright misconduct, namely any practice involving fabrication, falsification, and plagiarism (FFP). Others are commonly addressed in the literature as practices situated in the “gray area” between responsible conduct of research and severe misconduct (ALLEA, 2017; NHMRC, 2018; Sacco et al., 2019). Our usage of QRP includes both the “gray area” and outright misconduct. We intentionally avoid a more fine-grained distinction for two reasons.

One is that definitions of misconduct are field-specific (Ravn & Sørensen 2021). For example, insufficient study of existing literature on a given topic and failure to cite sources is considered a questionable practice for humanities scholars; while fishing for data without a well-developed research plan was considered to go against sound research practices by medical scientists.

Another reason is that even within particular fields, the boundary between misconduct and problematic but still acceptable practices changes over time. Research on the so-called replication crisis in fields like psychology and biomedicine suggests that certain practices of selective data use was previously rather widespread (Ioannidis, 2005; Open Science Collaboration, 2015). It is only recently that they tend to be seen as an outright ethical transgression and as such have become subject to more formalized prevent policies by scientific journals, for example through mandatory submission of raw data and different forms of preregistration of research (Denworth, 2019).

## Data & Methods

The data used in this paper stems from 30 focus group interviews carried out in eight different European countries in 2019–2020 as part of EC-funded project “Standard Operating Procedures for Research Integrity” (SOPs4RI, Grant Agreement no. 824481).^1^ The interviews sought to prioritize RI themes or areas that were lacking in guidelines and/or standard operating procedures through researchers’ and stakeholders’ input. The participants were from four main areas of research (humanities, social science, natural science incl. technical science, and medical science incl. biomedicine) and comprised 147 researchers of different levels of seniority and stakeholders both from RPOs and RFOs.

Recruitment was based on a number of sampling criteria to ensure overall variation in research areas and disciplines, as well as in stakeholder representation. For both the researcher-only groups (targeting RPOs) and the mixed groups (targeting RFOs), sample homogeneity was employed with regard to area of research; while the RPO groups were composed of researchers with shared methodological and epistemic approaches. Seeking to enhance representation and diversity, and to introduce heterogeneity into each group, the following criteria were also applied: senior/permanent position holders and junior researchers/non-permanent position holders; stakeholders from high-level management position in an RFO and from a research integrity office; the representation of two to three different disciplines; and gender balance amongst others (Table 1). The focus group study applied a purposeful sampling strategy (Patton, 1990) based on the criteria mentioned above, as well as a snowball/chain sampling. This strategy was supplemented by choosing participants from institutional webpages and inviting them by e-mail (Sørensen et al., 2021).

**Table 1:** Composition of groups distributed on gender & academic level Sørensen et al., (2021)

Main areas of research	No. of groups	Participants	Female	Male	Prof./prof. MSO/prof. emeritus	Associate prof./senior researchers	Assistant prof./post docs/PhDs/ junior researchers
Humanities	7	34	18	16	8	10	14
Social sciences	7	32	20	12	2	12	14
Natural science	8	42	17	25	12	8	12
Medical science	8	39	22	17	10	15	8
Total	30	147	77	70	32	45	48

Non-researcher participants are not listed separately. For a more detailed overview of the composition of the groups as well as acceptance rate, please see Table S4 in Sørensen et al. (2021)

The interview’s design consisted of three parts: a set of open questions, a discussion on selected RI topics, and a sorting exercise where topics were ranked between “very important”, “somewhat important”, and “of none or minimal importance”. The topics were identified from a three-round Delphi consultation of 69 research policy experts and institutional leaders on RI promotion plans. The number of topics to be discussed and ranked were nine for the RPO groups, and eleven for RFO groups.

The nine topics for RPO groups were: education and training in RI; responsible supervision and mentoring; dealing with breaches of RI; research ethics structures; data practices and management; declaration of competing interests; research environment; publication and communication; and collaborative research among RPOs. The eleven topics ranked by the RFO groups were: dealing with breaches of RI; declaration of competing interests; funders’ expectations of RPOs; selection and evaluation of proposals; research ethic structures; collaboration with funded projects; monitoring of funded applications; updating and implementing RI policy; independence; publication and communication; and intellectual property issues. For a full report on the interviews and its results please see SOPs4RI’s D5.2: Report on the Results of the Focus Group Interviews  (Sørensen et al., 2020).

Ethical approval for this research was obtained from the Research Ethics Committee at Aarhus University (ref. no 2019-0015957). All interviews were performed, recorded, and transcribed in English. Transcribed interviews were coded in the software program NVivo (ver. 12). For this paper, the coding process made use of an explorative approach, where the themes of QRPs and social uncertainty in academic careers emerged through an inductive coding procedure. The data was coded by one of the authors but discussed by both of them.

All relevant documents and reports, as well as all transcripts from the focus group interviews (in an anonymized form) can be accessed via the study’s OSF page: https://doi.org/10.17605/OSF.IO/E9U8T (Sørensen et al., 2023).

## Empirical Analysis

### Cutting Corners

During the interviews, topics that were not specifically addressed through the project’s questions kept resurfacing. In particular, the participants shared experiences of individual strategies that seek to reconcile tensions between epistemic and social uncertainties surrounding scientific research. One main concern is to optimize the use of available research time in the sense of prioritizing certain activities over others. As one researcher mentioned “Time is very important, if we have time there is no reason to go towards misconduct.” (professor, public health). One strategy employed to deal with time constraints consists in avoiding analytical steps in a given project or experiment that might in the short term increase the epistemic uncertainty researchers have to deal with before results can be published—for example by raising questions about the representativity or robustness of earlier results—although they could principally help strengthen the robustness of research. What might otherwise be referred to as sloppiness thus has a quite specific function, namely that of preventing a complication that would require further work to be resolved before researchers are able to publish results. Various respondents referred to this tactic as a form of “cutting corners” (researcher group, humanities).

The following quotes illustrate it through a variety of examples. A first example focuses on “cutting corners” as a tactic employed by individuals in the sciences in the context of a time-constrained project, where researchers skip additional analysis of a dataset to avoid complicating the results that have been obtained by that point (and thus potentially delay their publishability):

there is sort of a time-pressure especially when your PhD contract is almost finished. So, maybe at some point, you don’t do this extra analysis, you don’t extra check these outputs. And so, I feel like maybe it’s the risk in my case or around me is more like sloppiness a little bit. Because, you just want to be very efficient with your time. (junior researcher, medical sciences)

A variant of this tactic—arguably more often found in the humanities and social sciences—is when researchers adopt ready-made interpretations of previous research on a topic from published literature, instead of reading and directly engaging with this literature or a primary source. This practice not only saves time by allowing researchers to essentially copy-paste text elements for literature review sections. It also preemptively reduces the interpretive work (and thus epistemic complexity) involved if the authors were to read the quoted literature in detail. One of our respondents described this practice as risking to perpetuate problematic interpretations of previous literature:

(...) it does happen that people only read the most recent articles and books. So there are cases where you’ll find something maybe in the 80s and somebody cited a book from the 60s and that book was cited in the 90s, and everybody’s only citing the book from the 90s. If you go back to the original book, you find well the author didn’t quite say that or the evidence isn’t as strong as they said. (assistant professor, humanities)

#### Data Practices in the Grayzone

Manipulating data is a strategy to reduce epistemic uncertainty in the most immediate way. This includes the outright systematic fabrication of datasets or parts of datasets, which is ultimately a form of simply lying about the actual epistemic uncertainty encountered in research (Fanelli, 2013; Levelt et al., 2012; Vogel, 2011). Some researchers see the fabrication of data as an “obvious” response to publication pressure: “The most important thing is to remove the pressure of publishing which leads to falsifying results” (professor, public health). Most researchers, however, recognize the practice of fabrication as wrong: “People know what the right behavior is.” (assistant professor, humanities); “(…) I mean, you know exactly what is wrong and not what is not wrong.” (researcher, mathematics).

By contrast, the various forms of “p-hacking”, i.e. the selective use of data in statistical analysis are usually seen as a gray area practice, and thus as more acceptable:

(...) I think fraud, real fraud is I think very rare, but you can make yourself believe that something is not p-hacking, whereas in the end it is. (associate professor, social sciences).

Yeah, p-hacking, and I guess really people would be tempted to go into the grey area. Maybe not outright fraud, but: "Can I remove an outlier still? Can I test this in another way? Coming up with hypotheses after the data has been gathered.” (assistant professor, social sciences)

Another strategy related to data is to produce “low quality papers” which can be quickly published and which allow researchers to be nominally successful in the sense of producing output—although not necessarily robust, transparent, and reproducible research results:

(…) many uhm the students now are not so worried about this integrity but more are worried about finding a job. So the argument is that if you don’t get a job soon, then you cannot do research and then you cannot be, you cannot apply uhm integrity tools. So the important thing for them is to get the job, and if this means to publish a lot of papers with uhm low quality that could be fine. And in some way you can understand this this thing. (researcher, natural sciences)

#### Research Ethics as a Box Ticking Exercise

Research is increasingly subject to institutional oversight to prevent misconduct, often through various bureaucratic measures. This includes for example the requirement to create data management plans (DMP) for research projects to ensure that raw data are made accessible and can be used to reproduce findings or for further research; or preregistration of research proposals before carrying them out, to prevent that researchers retrospectively change their research design to fit spurious results. The principle thus is to impose new formal constraints on research practices to make them tractable for external scrutiny. Yet a risk for research ethics emerges precisely from the high degree of formalization of such quasi-regulatory practices. On one hand, formalization means additional documentation work for researchers that competes with other demands on their already scarce time, thus exacerbating social uncertainty. Simultaneously, formalization also creates the temptation for researchers to meet requirements in a merely symbolic way, namely by “ticking a box” and complying to formalized minimal requirements, but without seriously engaging with the underlying ethical considerations.

Yes, I’m not really fond of the fact that institutions should for every different funding channel have all these kinds of things in place because then you just create another tick box because we already have to apply to a lot of things and you don’t really create a change in mentality, which is more important, I think, than having all, then you can prove, you have a document in place because I don’t think you change mentality with only documents. (research coordinator)

A concrete example described in our material is part of grant writing practices. Grant applications nowadays often require researchers to submit sometimes very detailed DMPs. As the researcher in the following quote explained, it is not uncommon to simply reuse documents from previous applications with only minor tweaking. On one hand, this can simply be seen as a practice to streamline the amount of work invested in competitive funding acquisition, itself a key source of social uncertainty in research. Yet at the same time, this form of symbolic compliance to ethics requirements where a DMP is reused without a careful review also undermines the very transparency and reproducibility that are the rationale for DMPs in the first place.

Because when we get asked to like write a data management plan, we all go back to the data management plans that we wrote for the last 6 applications and just you know change some names. That’s not responsible research necessarily, but it’s kind of the only thing that you can do in the time that you’ve got. (...) I don’t think it’s good practice and it’s bad practice I think in our case often has emerged, or poor practice has emerged, because the constraints are so high and the rewards don’t always feel that great either you know. It’s a terrible balance. (associate researcher, humanities)

#### Avoiding Trouble

Another time-optimization strategy is to focus solely on one’s research and intentionally avoid uncomfortable confrontations with colleagues about questionable research or related academic practices. Several respondents reported that they had observed problematic integrity issues on various occasions and chose not to report them. We frame this as a measure to avoid additional social uncertainty because it may result in threatening the position and standing of whistleblowing researchers in their institution, thus threatening also the practical conditions to pursue a career in the long term:

(...)it is also not going to help if people who see something, and they are like: Yeah, but it will in the end cost me my job and the person that did the wrongdoing might still, you know like (…). (member of compliance review committee, private research institution).

As a researcher mentioned:

(…) one of the biggest hurdles has been the time element because if there’s a PhD student who has an issue with their supervisor, for example, and the meeting is once a year, and they miss the meeting for reviewing these cases, that’s a third, a quarter of the time for their whole contract and so it feels like, I know from speaking with several of these PhDs, that that feels like too much of a time commitment and they should just tolerate these terrible circumstances, perhaps, just to get by. (researcher, humanities)

Such strategies of ignoring problematic behavior is perceived as particularly advisable by researchers in precarious contract situations, including guest, visiting, and junior researchers. As the following testimony shows, such differences in status make it risky for precarious researchers to raise issues related to RI in their workplace, especially where they involve more established colleagues:

And I'm sure if I had a permanent position now, then I would be a lot braver to speak out about a lot of things which are bothering me...Uhm and I suppose I’m also quite lucky that I don’t feel my personal integrity has been compromised. But I know colleagues who have for instance been involved in co-editing books, which are allegedly peer reviewed where they never have been peer reviewed and these kinds of practices which I really don’t find acceptable, but if I was asked "would you co-edit this book?" and then I knew that was going on, I would still find it hard to speak out. (assistant professor, humanities)

Sometimes, such risk-avoidance is in fact perceived as opportunistic. Another interviewee noted that especially visiting researchers are more likely to ignore problematic behavior while selectively taking advantage of what an institution has to offer. The problem here seems to be improper embedding of researchers in the host institution, which makes it easier for them to shirk ethical responsibilities.

[Guest researchers] just put on the name of an institution, use the resources of an institution, but they are not accountable to the institution. (member of the Research Ethics committee).

A more general variant of what we propose to subsume under the heading of “avoiding trouble” is avoidance of ethics training or other departmental gatherings (researcher, humanities) meant to sensitize researchers for ethical issues. Our focus groups respondents reported that researchers commonly try to free up their schedules by avoiding obligations that are not immediately relevant to publishing or other explicitly rewarded activities like grant writing. Ethics-related information campaigns, too, are commonly seen as distractions by many academics, thus putting a particular question mark behind the notion that misconduct can be conceptualized as resulting from an information deficit. It rather appears that the deficit itself is part of an effort to reconcile already significant competing constraints on research practice.

#### Authorship & Grant Writing Practices

Another widespread tactic to reduce the social uncertainty that comes with a highly competitive job market is related to authorship practices. In the following, a respondent describes a practice that is otherwise known as ‘gift authorship’, i.e. a *quid pro quo* approach to sharing publication credits that reduces the risk of ending up without accountable results from a research project, even where the underlying work was not really collaborative. While not necessarily reproachable in ethical terms, the practice alters the meaning of authorship, contradicts what many guidelines define as good scientific practice, and was highlighted as a gray area behavior for most disciplines in Ravn & Sørensen (2021):

I think the authorship is definitely also something that has (unclear), because there is also external pressure. You need to have many publications and many citations, and so the system reacts to external forces. So if you look at the average number of co-authors on our papers, it’s just going up and up and up, because people are just, and they say "you’ve put me on you paper, I’ll put you on my paper". (associate professors, natural sciences)

Another concern is the way in which funding for scientific research operates on the level of grant writing practices. A major source of social uncertainty is the hesitation of what type of research might appeal to funding bodies. Scientists feel that due to a lack of basic funding they must dedicate a large proportion of their time chasing grants with a low rate of success (research associate, clinical science; professor and senior researcher, clinical science). A strategy commonly used when submitting proposals is to “accommodate” one’s own research to topics that are being more likely to be funded, whether it benefits the larger line of research or not (Hackett, 1987; Laudel, 2006; Leisyte & Enders, 2011):

(...) these massive grants within [country] work, the way the application procedure works, is that you’re dealing with a committee of, ultimately, people who are not in your field. So that is a way, in which, then big projects with, for lack of a better way of saying it, you know, certain buzzwords which kind of form a certain common denominator of what this committee of eight people, who are not directly in your field but in a related field, agree on, choose to fund. But then there’s all these ways in which you can also push that system. (researcher, humanities)

Respondents also described another way of reducing the uncertainty involved in trying to get funded by explicitly catering to the interests of commercial partners, sometimes with a potential for undermining transparency and the integrity of research. A researcher shared an exchange with a colleague that illustrates how in certain disciplines it is encouraged to “fool the system”:

[name] who is the head of the [academy] used to work in my department and I worked with [them] and [they] said, if you can’t figure out a way to get [multinational conglomerate corporation], the company, to sponsor your research, then you’re not creative enough. And then I think, well... you know, how should I make [the specific type of literary criticism practiced by the speaker] relevant to [a multinational conglomerate corporation]? (researcher, humanities)

## Discussion

In this paper we have primarily conceptualized QRPs as attempts to reconcile epistemic and social forms of uncertainty in knowledge production. This does not mean that we are trying to relativize or excuse misconduct. The conceptualization is rather meant to offer a more fine-grained analytical perspective on what exactly it is that researchers want to accomplish by choosing particular forms of misconduct, thus going beyond the descriptive categorization of misconduct (Fanelli, 2011), or observations of motivated reasoning to justify QRPs (Bruton et al., 2020; Mazar and Arely, 2015; Sacco et al., 2019). This in turn makes it possible to offer more specific possibilities for discouraging these behaviors.

The QRPs that surfaced during the focus group interviews shed a light on how social uncertainties are tightly coupled to epistemic ones. The majority of them are rooted in how science funding is organized, the erosion of public funding to science, and the emphasis of research management on quantified decision-making (Pardo-Guerra, 2022). This in turn translates to specific working conditions such as prolonged sequences of temporary contracts, a lack of plannability in career development, and a higher workload due to erosion of resources all around. In the current context where efficiency is seen as a proxy for quality, scientific career prospects depend on evaluations that focus strongly on metrics on the level of individuals, mainly the number of publications in prestigious journals and citations. It is worth noting that by relying so heavily on metrics, funders and managers seem to also apply one of the strategies mentioned by researchers, namely that of cutting corners in assessing the quality of someone’s work.

We are currently in the midst of a widespread debate about scientific misconduct (Horbach & Halffman, 2017; Valkenburg et al., 2021). While some fields are more often mentioned in this context than others, the discussion pops up across the natural, medical, and social sciences, as well as the humanities (Ravn & Sørensen, 2021). Interestingly, universities as well as policy making bodies increasingly react to this discourse through normative approaches on an institutional level. Some examples are the initiatives by the European Commission to promote the adoption of RI codes across European universities (ALLEA, 2017) and the implementation of RI promotion plans by Horizon Europe participants (HE Model Grant Agreement 2021, HE Programme Guide, 2022). The approach is based on the assumption that misconduct is to some degree the result of an information deficit and can be thus effectively fought through providing information. Although this approach is helpful to regulate specific aspects, such as the definition of authorship, it ignores underlying problems like the erosion of public funding and how this puts pressure on the long term planning of research and of academic careers.

Under the various headings, we discussed QRPs that are geared to hide or reduce scientific uncertainty, since this is directly coupled to the risk of ending up without publications at the end of a fixed-term contract, and hence jeopardizes a career. We would call on disciplinary communities and institutions to not simply meet this problem with guidelines about integrity, which effectively can create simply another constraint on current ways to reconcile uncertainties.

We rather recommend efforts to build publication cultures where scientific uncertainties are not primarily a problem of individual scientists, but where such uncertainty can be acknowledged as a resource for scientific knowledge production on a collective level. This could mean doubling down on ongoing reform initiatives such as preregistration (provided that it also reduces the stigma of publishing “boring” data), journals and publishing platforms dedicated to negative results (Bruton et al., 2020; Matosin et al., 2014), platforms dedicated to the publication of data (such as for digital humanities), but more generally also rethinking publication acceptance criteria in journals. Perhaps it also means pushing for alternative publishing formats such as preprints, where publishability is not determined by gatekeepers, but where peer review is provided after making a manuscript publicly accessible (although preprints may of course create new opportunities for misconduct in their own right) (eLife, 2022; Polka et al., 2022). Most importantly, we believe that such change in publication cultures cannot be induced top down from an institutional level, but rather has to emerge at least partly from within academic communities. Another recommendation is for institutions to change the focus of evaluations from metrics to more qualitative methods. Responsible research evaluation where a broader variety of activities and impacts are considered can further aid in reconciling social and scientific uncertainties in ways that accommodate a broader range of research practices (Rijcke et al., 2019; Ioannidis & Khoury, 2014).

Throughout the focus groups interviews, participants shared their skepticism towards the potential of guidelines alone to induce change at research institutions, seeing them instead as yet another bureaucratic tick-box exercise. To avoid this development, they proposed some recommendations. One of the suggestions was the need for spaces where scientists of different levels could discuss the issues and challenges that surface during research: “it’s more about being open to saying and open to debating, what are good practices and what are bad practices and how do we all practice better?” (assistant professor, humanities). They also mentioned the need for counselors and advisors for researchers to consult and confide in (assistant professor, social sciences). Both those options are more costly than trying to solve issues through the release of documents that must be complied with. Even though the latter need to be revised and updated, the former require resource investment in the shape of dedicated personnel, whether full or part-time, and that institutions strife to reduce hierarchies creating an equitable working environment where issues can be openly raised without fear of retribution (Ahmed, 2021). The exchanges and communication that would arise from such opportunities would be highly relevant for building a healthy research environment where RI is part and parcel of the system.

Our final recommendation is related to job precarity. At the root of many of the QRPs are the working conditions, as highlighted elsewhere by testimonies from researchers themselves and policy papers (Hesselberg, 2020; OECD, 2021). Institutions need to foster fair, equitable, and healthy environments. Research organizations must be realistic and fair both towards their employees and funders; funders, especially the public ones, need to recognize the disparity between a discourse on a healthy work environment and the working conditions on the floor. Researchers and staff across the board can also push for change by organizing and demanding better working conditions. In this last step it is important that those researchers in non-precarious conditions support their precarious colleagues. Given that precarity not only affects researchers but is found across the board in RPOs and RFOS, the different communities involved can seek to develop commonalities to transform the working environment (Lorey, 2015).

Our recommendations should not be seen as a dismissal of guidelines concerning RI. Such documents are relevant as points of reference that researchers and staff can appeal to. However, as an “easy fix” to RI issues that do not require any structural reforms in how science is organized, they leave unaddressed current underlying obstacles for a healthy research environment. Uncertainties, both epistemic and social, are a permanent feature of scientific research. The manner in which research is currently organized, individualizing its social risks for the sake of efficiency and cost effectiveness for institutions, is not only a risk for RI but also stifles creativity, as highlighted by the following quote:

“I think also we miss an environment that in some way promotes the creativity, the rigour of the research, the tranparence of the research. I think we should (...) proactively look for or create and environment that improves the the quality of the research in general. Not just the productivity, but the quality of the research.” (researcher, natural sciences)

The majority of researchers are aware of what good research entails. As some quotes from the analysis show, they are not proud of gray area breaches but they accept them as strategies to survive in a specific system. Collectivizing the costs of uncertainty, building more inclusive publication cultures, applying responsible evaluation methods, providing more long-term resources for an open and transparent culture, and working to de-hierarchize institutions, need to go alongside the release of guidelines and other documents.

## Data Availability

All relevant documents and reports related to the focus group study can be accessed via the study’s OSF page: https://doi.org/10.17605/OSF.IO/E9U8T. The focus group invitation letter included a link to the privacy policy specifying the procedures for data management and privacy in compliance with the General Data Protection Regulation (GDPR).

## Footnotes

1 The focus group interviews were carried out in Denmark, Spain, the Netherlands, Germany, Belgium, Croatia, Italy, and Greece. 22 of them were conducted as face-to-face interviews, while the last eight had to be carried out online because of the COVID-19 pandemic.
